# The Importance of Drains for the Larval Development of Lymphatic Filariasis and Malaria Vectors in Dar es Salaam, United Republic of Tanzania

**DOI:** 10.1371/journal.pntd.0000693

**Published:** 2010-05-25

**Authors:** Marcia C. Castro, Shogo Kanamori, Khadija Kannady, Sigsbert Mkude, Gerry F. Killeen, Ulrike Fillinger

**Affiliations:** 1 Department of Global Health and Population, Harvard School of Public Health, Boston, Massachusetts, United States of America; 2 Department of International Community Health, Graduate School of Medicine, The University of Tokyo, Tokyo, Japan; 3 City Medical Office of Health, Dar es Salaam City Council, Dar es Salaam, United Republic of Tanzania; 4 National Malaria Control Program, Dar es Salaam, United Republic of Tanzania; 5 Coordination Office, Ifakara Health Institute, Dar es Salaam, United Republic of Tanzania; 6 Vector Group, Liverpool School of Tropical Medicine, Liverpool, United Kingdom; 7 Disease Control and Vector Biology Unit, Department of Infectious and Tropical Diseases, London School of Hygiene and Tropical Medicine, London, United Kingdom; University of Copenhagen, Denmark

## Abstract

**Background:**

Dar es Salaam has an extensive drain network, mostly with inadequate water flow, blocked by waste, causing flooding after rainfall. The presence of *Anopheles* and *Culex* larvae is common, which is likely to impact the transmission of lymphatic filariasis and malaria by the resulting adult mosquito populations. However, the importance of drains as larval habitats remains unknown.

**Methodology:**

Data on mosquito larval habitats routinely collected by the Urban Malaria Control Program (UMCP) and a special drain survey conducted in 2006 were used to obtain a typology of habitats. Focusing on drains, logistic regression was used to evaluate potential factors impacting the presence of mosquito larvae. Spatial variation in the proportion of habitats that contained larvae was assessed through the local Moran's I indicator of spatial association.

**Principal Findings:**

More than 70% of larval habitats in Dar es Salaam were human-made. Aquatic habitats associated with agriculture had the highest proportion of *Anopheles* larvae presence and the second highest of *Culex* larvae presence. However, the majority of aquatic habitats were drains (42%), and therefore, 43% (1,364/3,149) of all culicine and 33% (320/976) of all anopheline positive habitats were drains. Compared with drains where water was flowing at normal velocity, the odds of finding *Anopheles* and *Culex* larvae were 8.8 and 6.3 (p<0.001) times larger, respectively, in drains with stagnant water. There was a positive association between vegetation and the presence of mosquito larvae (p<0.001). The proportion of habitats with mosquito larvae was spatially correlated.

**Conclusion:**

Restoring and maintaining drains in Dar es Salaam has the potential to eliminate more than 40% of all potential mosquito larval habitats that are currently treated with larvicides by the UMCP. The importance of human-made larval habitats for both lymphatic filariasis and malaria vectors underscores the need for a synergy between on-going control efforts of those diseases.

## Author Summary

Lymphatic filariasis and malaria are endemic in Dar es Salaam and partially share common vectors, but no synergy exists between their control programs. The vast majority of mosquito breeding habitats in the city is human-made, and therefore could be mitigated through the implementation of a community-based vector control program accompanied by an educational campaign to sensitize the population and promote behavior change. Of particular importance are clogged drains and ditches with stagnant water, which are the most common larval habitat in the city. This problem could be addressed by an environmental management intervention in order to restore and maintain the functionality of drains. Such intervention can potentially contribute to reduce vector-borne disease transmission, but also promote an overall healthier environment, particularly improving the situation of those living under slum conditions, as proposed by the Millennium Development Goal 7. A synergy between efforts to control lymphatic filariasis and malaria, identifying common strategies, combining monitoring activities, optimizing the use of limited financial resources, and carefully evaluating the cost-effectiveness of the joint venture would not only contribute to current goals of lymphatic filariasis and malaria elimination, but also provide important lessons for future integrated control efforts.

## Introduction

According to the 2004 update of the Global Burden of Diseases (GBD) [1], 44% of the disease burden in the United Republic of Tanzania (as measured by disability-adjusted life years – DALYs) was due to infectious and parasitic diseases. Among those diseases, malaria carried the largest burden, 20%, and neglected tropical diseases accounted for 6%, half of which is attributed to lymphatic filariasis (LF). The estimated number of clinical malaria cases in the United Republic of Tanzania ranges between 14 and 19 million per year, and the estimated number of deaths between 100,000 and 125,000, of which approximately 80,000 are children under the age of 5 years [2]. LF is a major cause of permanent and long-term disability [3], [4]. It is endemic in all regions of the United Republic of Tanzania, with higher antigenemia levels (up to 45–60%) observed along the coast, and lower levels in the western portion of the country. It is estimated that 6 million people are infected with the debilitating manifestation of LF [5], which makes the United Republic of Tanzania the country with third highest prevalence of LF in sub-Saharan Africa [6].

Both malaria and LF are mosquito-borne diseases. Afro-tropical LF and malaria vectors show a high ecological plasticity utilizing a broad range of aquatic larval habitats [7]–[12]. Proper understanding of the typology (type, prevalence, and seasonality) of these habitats in a particular location is crucial for the adequate planning of vector control interventions. While the typology is often related to patterns of land use, local ecology, and human behavior, adaptation of mosquitoes to the characteristics of fast growing cities (e.g., mosquito larval development in habitats organically polluted by rotting vegetation or human feces) poses additional challenges for vector control efforts in urban areas [7], [8], [13]. Indeed, the current pace and pattern of urban growth has no precedents in human history. In 2008, for the first time, the majority of the world's population was living in urban areas. In Africa, the urban population is likely to double between 2000 and 2030, and it is estimated that more than half of Africans will live in urban areas by 2030, most in poverty. Currently, approximately 72% of the urban population in sub-Saharan Africa lives under slum conditions [14], [15]. This rapid urbanization alters the dynamics of mosquito-borne disease transmission, with significant effects on disease-associated morbidity and mortality, which in turn has important implications for disease control [16]–[18].

Fast urban growth often challenges government's ability to provide resources and to properly invest in urban planning. As a result, unplanned and unserviced settlements abound, characterized by lack of sanitation and drinking water, precarious housing, overcrowding, unpaved roads, and inefficient or inexistent solid waste collection [13]. In addition, it is common to observe clogged drains and ditches with stagnant water, particularly in those unplanned settlements, and the practice of urban agriculture is widespread [19]. These conditions pose serious environmental challenges [19], but also bring about many public health challenges, such as an increase in cholera risk [20] and the proliferation of man-made aquatic habitats suitable for disease vectors breeding [17], [21]–[24], to name a few.

Historically, environmental manipulation and modification of potential mosquito larval habitats by engineering works (e.g., drainage and filling) [25] to promote environmental management (EM) [26] were successfully adopted in both urban and rural settings [27], [28], mainly prior to World War II and the development and large-scale use of the insecticide DDT. Many important endeavors, such as the construction of the Panama Canal [29], [30], copper mining in Zambia [31], [32], rubber production in Malaysia [33], and the sanitation of Rio de Janeiro city, Brazil [34], [35], were successfully accomplished through EM efforts. In addition, many cities under colonial rule witnessed EM activities for disease prevention, including Dar es Salaam in the United Republic of Tanzania. EM efforts were initiated under the German rule, intensified under the British rule, and successfully continued after independence [36]–[45]. However, EM activities suffered a major setback in 1972, when adverse economic conditions resulted in deterioration of the national health system. Maintenance of drains was nonexistent; water flow was blocked by silt, vegetation, and waste, favoring the occurrence of flooding after the rains, and offering ideal conditions for mosquito breeding inside and in the immediate surroundings of drains [46].

Currently, Dar es Salaam still faces similar problems, augmented by the pace and pattern of urban growth, particularly since the early 1980s [47]. Dar es Salaam is among the world's ten large cities with fastest growth, and its population is expected to double between 2005 and 2020 [48]. In 2007, 29% of the urban population of the United Republic of Tanzania lived in Dar es Salaam. Approximately 65% of the households in Dar es Salaam are located in informal areas, living under slum conditions as defined by the United Nations Human Settlements Programme (UN-HABITAT), and therefore lacking one or more of the following conditions: access to an improved drinking water source, access to improved sanitation facilities, sufficient living area, durable housing in a non-hazardous location, and security of tenure [48], [49]. Rapid and unplanned urban growth have created areas with precarious infrastructure and inefficient solid waste collection [8], [45], [50]. According to reports of the Dar es Salaam City Council, approximately 50% of all refuse daily generated in the city is not collected, and a large portion eventually finds its way into drains and rivers.

A small-scale urban malaria control effort attempted in Dar es Salaam during 1986–1993 identified drains as important sources of *Anopheles* larval development [45]. In addition, a survey conducted in 2006–2007 in the City indicated that, on average, 21% of the drains contained immature forms of *Anopheles* and *Culex* mosquitoes throughout the year [51]. Both vectors are of public health importance: in Dar es Salaam, three species of *Anopheles* were identified as malaria vectors, namely *An. gambiae s.l.*, *An. funestus* and *An. coustani* [52], while LF is transmitted by *Culex quinquefasciatus*, *An. gambiae s.l.*, and *An. funestus* [53], [54]. Yet, the actual importance of drains relative to other breeding habitats in the city remains unknown.

Here, we analyze the typology of mosquito breeding habitats in Dar es Salaam, to assess the importance of drains as a source of larval habitats for vectors of LF and malaria. We examine the most common characteristics of drains that were associated with the presence of *Anopheles* and *Culex* larvae and discuss opportunities for a coordinated effort of vector control that could foster a synergy between current programs to control LF and malaria in Dar es Salaam.

## Materials and Methods

### Study area

Dar es Salaam is the largest city and *de facto* capital of the United Republic of Tanzania, located along the shores of the Indian Ocean. Administratively, the city comprises three municipalities – Ilala, Kinondoni and Temeke – and is divided into 73 wards, 51 of those considered to be urban, according to the National Bureau of Statistics. Wards are further divided into smaller neighborhood units called *mitaa* (a Kiswahili word for street, written in the singular form as *mtaa*) [55]. Each *mtaa* is subdivided into ten-cell units (TCU), or clusters of approximately 10–20 houses, although some TCUs contain a much larger number of houses.

**Figure 1:**
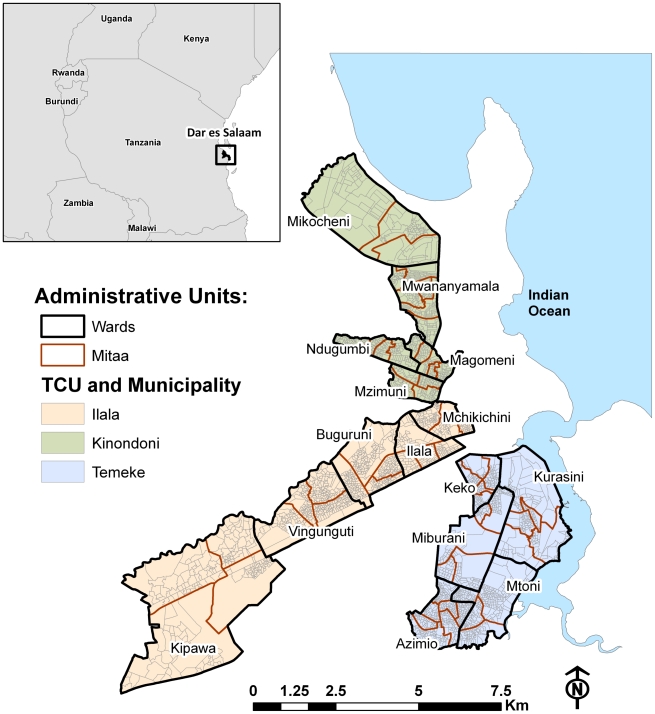
Study area and administrative units in Dar es Salaam, Tanzania. Administratively, Dar es Salaam comprises three municipalities – Ilala, Kinondoni and Temeke – and is divided into 73 wards (22 in Ilala, 27 in Kinondoni, and 24 in Temeke), classified by the Tanzania National Bureau of Statistics (NBS) as urban, rural or mixed. The wards are further divided into smaller areal units called *mitaa*, which are subdivided into ten-cell units (TCU), the smallest administrative unit in the city. The map highlights the 15 wards included in the study (5 in each municipality), which comprise the targeted area for an urban malaria control effort.

Our study focused on 15 city wards that comprise 56 km^2^ (Figure 1) and a population of more than 610,000 people. Since 2004, that area has been targeted with a large operational community- based Urban Malaria Control Programme (UMCP) [56], which included routine mapping and surveillance of mosquito breeding habitats. The UMCP commenced weekly larvicide application in March 2006 in three out of the 15 wards, scaled-up to nine wards in May 2007, and to all 15 wards in April 2008. The study area was also covered by a drain assessment survey conducted in 2006–2007, which gathered information on varied characteristics of drains, including larval presence [51].

### Routine larval habitat mapping

Information on characteristics of mosquito breeding habitats during 2005–2007 was obtained from the UMCP routine larval habitat surveys, which have been described in detail elsewhere [56]. Briefly, all habitats which were open to sunlight (referred to as ‘open habitats’) in the targeted area were surveyed for mosquito larvae by dipping [57] and the presence or absence of anopheline and culicine larva recorded. The UMCP priority is malaria vector control, therefore sanitation structures, such as pit latrines, soakage pits, and container type habitats which are closed to the sun (referred to as ‘closed habitats’) and produce large numbers of culicines, but no *Anopheles* [58], [59] mosquitoes were not included in the surveys. Each habitat was assigned a unique identifier and followed-up weekly. Habitat characteristics recorded were: habitat type, habitat size in perimeter, presence of water, and occurrence of emergent plants inside the water. Only aquatic habitats (containing water) were included in this study, and they were grouped into ten categories: (1) puddles, (2) swampy areas, (3) mangrove swamps/saltwater marshes, (4) drains, (5) streams/river beds, (6) construction (including construction pits, foundations, man-made holes), (7) water storage containers, (8) agriculture (including rice paddies, ridge and furrow agriculture, and other habitat associated with agriculture), (9) ponds, and (10) others (e.g., spring and seepage).

Although data were collected on a weekly basis, to date only information from one week in every 4-week period was stored in digital format. Therefore, survey data were available for 13 weeks per year, which comprised a representative sample of the annual typology of breeding habitats in the city. All data collected between 2005 and 2007 were aggregated at the TCU level by month, year and habitat type. Since our goal was to determine the importance of drains as breeding habitats for disease vectors, and not to evaluate the use of larviciding introduced by the UMCP, we excluded from the analysis all wards covered with this intervention in 2006 and 2007. We evaluated the proportion of aquatic habitats that contained *Anopheles* and/or *Culex* larvae by habitat type. In addition, among those aquatic habitats that did contain larvae, we assessed which were the most common types.

### Drain assessment survey

**Figure 2:**
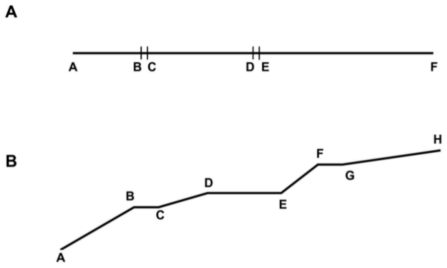
Sketches of drain segments that compose one unique surveyed drain. The Figure shows two hypothetical drains, indicating how different segments were identified and surveyed during the Drain Assessment Survey. **A**. The drain follows the same linear direction throughout its entire extension, but five drain segments are identified: three are open (AB, CD, and EF) and two are covered – car and/or pedestrian passage (BC and DE). **B**. The drain follows local areal characteristics, and each change in direction corresponds to a unique drain segment.

During May 2006 and March 2007 we conducted a survey in the study area to assess the physical characteristics of the drains (structure, material, dimensions, and geographic coordinates) and their current conditions (undergoing maintenance activities, presence of waste and/or vegetation, water flow, accessibility, history of flooding, and presence of *Anopheles* and/or *Culex* larvae) [51]. Each drain was surveyed once for the same set of parameters in different segments, which represent sections with similar direction or separated by covered structures (e.g., car and/or pedestrian passage; Figure 2). City cadastral maps (available at 1∶2,500 scale) were used to locate drains. These maps were produced based on 1992 information, and since then have not been updated. Survey-related field work included (i) locating existing drains in the cadastral maps, or sketching the drain if it was built after 1992; (ii) collecting geographic coordinates at an accuracy level of approximately 5–8 m by the use of a global positioning system (GPS) receiver (Garmin eTrex® H; Olathe, KS, USA); (iii) filling out a data collection sheet for each drain segment; (iv) taking pictures with a digital camera to document drain conditions; and (iv) checking for the presence of larvae using a mosquito dipper (using the same protocol adopted in the UMCP larval habitat survey). A total of 338 drains were surveyed, comprising 3,272 drain segments and 107.6 km in extension.

### Spatial information and rainfall data

We used the geographic coordinates of surveyed drain segments to create a spatial database. Points representing each segment were connected to create a polyline for each surveyed drain. We also utilized a 2002 Quickbird satellite image to validate the location of drains not represented in the cadastral maps. Habitat-related variables were arranged in a spatial database with TCUs as the spatial unit of analysis. TCU maps were obtained through participatory mapping as part of the UMCP activities [55].

Dar es Salaam has a hot and humid tropical climate with two rainy seasons: an intense one observed during the months of March, April, and May, and a milder one occurring in November and December. To account for this seasonality, monthly rainfall data were provided by the Meteorological Agency, and a categorical variable created to represent dry and wet seasons. Dry months were January, February, June, July, August, and September; wet months included March, April, May, October, November, and December.

### Spatial analysis

Spatial variation in the proportion of aquatic habitats that contained *Anopheles* and/or *Culex* larvae (as reported by the UMCP larval habitat survey data) was assessed through the use of the local Moran's I indicator of spatial association [60]. Significant clusters were identified utilizing a first-order queen neighborhood definition. All results were corrected for multiple testing utilizing the false discovery rate control procedure [61], [62]. Mapping was performed in ArcMap (ESRI, Redlands, CA, USA), and spatial analyses were conducted in GeoDA [63].

### Habitat characterization and potential determinants for *Anopheles* and *Culex* breeding

We analyzed the UMCP larval habitat survey data to obtain a typology of aquatic habitats in the study area and to assess their seasonal variation due to rainfall. Focusing on drains, we used the drain assessment survey to evaluate potential factors impacting the presence of larvae in these habitats. We fitted two separate logistic regression models. In the first model, the outcome variable indicates the presence of *Anopheles* larvae in surveyed drain segments. In the second model, the outcome variable indicates the presence of *Culex* larvae in surveyed drain segments. In both models we included independent variables that showed a significant effect in univariate analysis. These independent variables were all binary and included: (i) presence of a human-made connection to the drain segment – these connections are often illegal and tend to leave exposed pipes inside the drain blocking the water flow; (ii) lined drain segment – lined drains are less likely to present erosion; (iii) located in residential areas; (iv) presence of waste materials; (v) drain segment contained vegetation; (vi) efforts to maintain the drain in good conditions were conducted regularly; (vii) drain segment often floods after the rains; (viii) presence of stagnant water; (ix) water flowing at low velocity; (x) drain segment easily accessible by trucks; (xi) drain segment constructed before 1992; and (xii) drain segment surveyed during the dry season. Finally, the first model included a binary variable indicating the presence of *Culex* larvae, and the second model included a binary variable indicating the presence of *Anopheles* larvae.

Model goodness-of-fit was assessed through the calculation of three diagnostics measures: (i) the area under the receiver operating characteristic (ROC) curve; (ii) the detection of extreme observations (Pearson residuals, standardized residuals, deviance, and Pregibon leverage); and (iii) the assessment of heteroscedasticity in the residuals (robust standard errors) [64]. All data were stored in databases prepared in Epi Info™ version 3 (Centers for Disease Control and Prevention; Atlanta, GA), or Microsoft Excel (Microsoft Corp.; Seattle, Washington). All statistical analyses were done using Stata/SE 9.2 (Stata Corp.; College Station, TX, USA).

## Results

**Figure 3:**
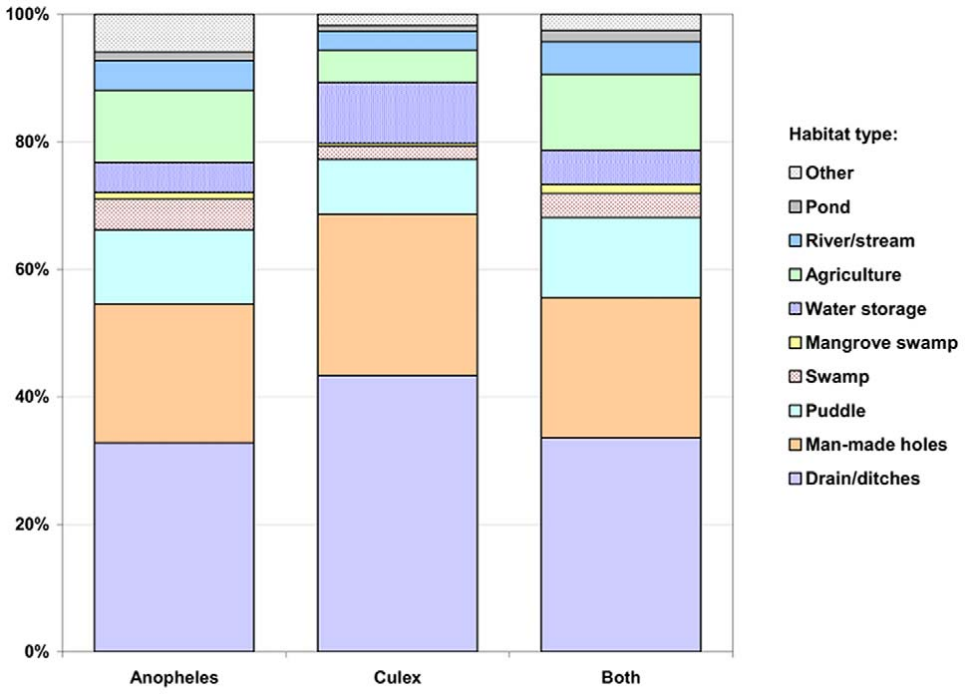
Percentage distribution of aquatic habitats that contained larvae by habitat type, 2005–2007. Data on aquatic habitats containing *Anopheles* or *Culex* larvae were retrieved from the Urban Malaria Control Program (UMCP) routine larval habitat survey.

**Figure 4:**
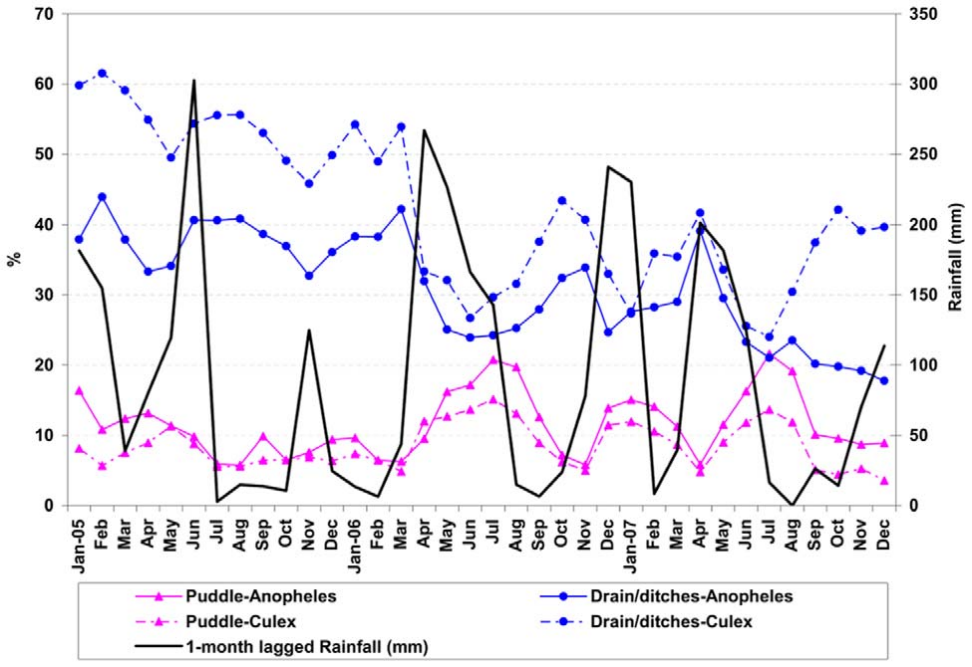
Monthly distribution of rainfall and percentage of drains and puddles that contained larvae, 2005–2007. Data on aquatic habitats by type were retrieved from the Urban Malaria Control Program (UMCP) routine larval habitat survey. Rainfall information was provided by the Tanzania Meteorological Agency, and was lagged by 1-month. Therefore, mosquito larval presence in a month is compared with the amount of rainfall in the previous month.

Between 2005 and 2007, on average 24,039 habitats were followed-up weekly, and 54% (12,888) contained water in one or more of the surveyed weeks (Table 1). On average, 8% of the aquatic habitats that were found per sampling occasion contained *Anopheles* larvae, 24% contained *Culex* larvae, and in 5% the concurrent presence of both anopheline and culicine larvae was observed. A detailed analysis by habitat type (Table 1) indicated that aquatic habitats associated with agriculture were proportionally most frequently colonized by *Anopheles* (33%), followed by ponds (25%) and habitats associated with rivers and streams (21%). In contrast, anophelines were, on average, found in only 6% of the drains. Overall, a higher proportion of habitats were colonized by culicine larvae than anopheline larvae. *Culex* larvae were, on average, present in 51% of the ponds, 48% of habitats associated with agriculture, 43% of habitats associated with rivers and streams, but in only 25% of the drains. Notably, the vast majority of the weekly surveyed aquatic habitats were drains (42%) and only 3% of the habitats were associated with agriculture. Therefore, 33% (320/976) of all anopheline positive habitats and 43% (1,364/3,149) of all culicine positive habitats found per weekly survey were drains and ditches, three and eight times more than anopheline and culicine positive agricultural sites, respectively (Table 1 and Figure 3). Construction pits, foundations, and other human-made holes were the second most common habitat types (Figure 3), and combined with drains accounted for more than half of the aquatic habitats positive for *Anopheles* larvae. This typology of larva-positive habitats showed seasonal variation due to rainfall, particularly for drains and puddles (Figure 4). Heavy rains increase the water level in drains, causing flooding in those with precarious conditions, and washing off waste materials. For a short period immediately after the rains the water flow can be restored, and the presence of larvae in the drain reduced. However, over time, waste eventually gets back into the drains, disturbing water flow and facilitating mosquito breeding. In contrast, puddles positive for larvae become more prominent after the rains, and some of these puddles often occur in the vicinity of drains after flooding.

**Table 1:** Average number of habitats surveyed weekly between 2005–2007.

Habitat type	Total habitats	Aquatic habitats	Habitats with larvae					
			*Anopheles*		*Culex*		Both *Anopheles* and *Culex*	
			Total	% with larvae	Total	% with larvae	Total	% with larvae
Puddle	4,721	1,519	114	7.5	270	17.8	85	5.6
Swamp	789	428	47	11.0	67	15.5	25	5.9
Mangrove swamp	60	51	10	19.5	12	23.4	9	18.3
Drain/ditches	8,198	5,405	320	5.9	1,364	25.2	226	4.2
Human-made holes	5,337	3,028	212	7.0	797	26.3	148	4.9
Water storage	2,983	1,518	46	3.0	301	19.8	36	2.4
Agriculture	1,047	334	110	33.0	160	47.9	80	24.0
River/stream	220	216	45	21.1	92	42.6	34	16.0
Pond	67	57	14	23.8	29	50.0	12	21.1
Other	617	332	58	17.3	57	17.1	17	5.1

**Figure 5:**
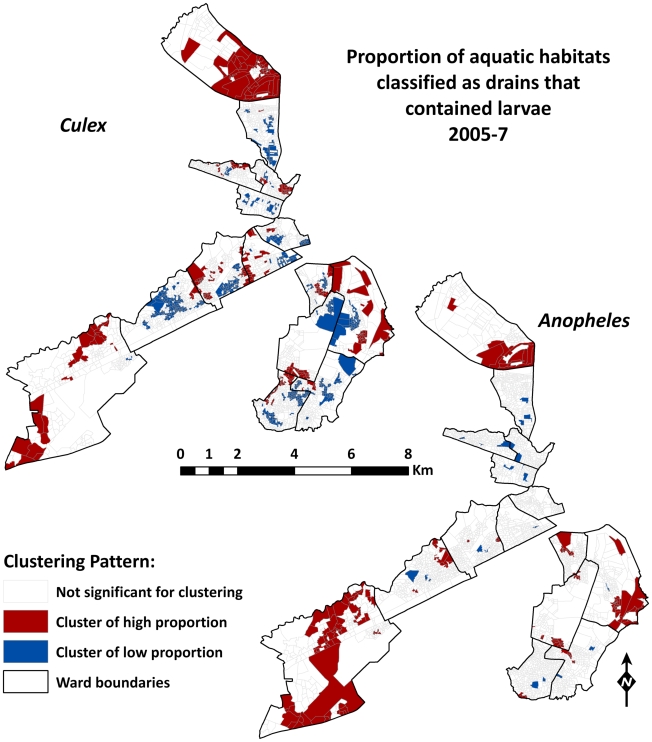
Clustering pattern in the proportion of aquatic habitats classified as drains that contained larvae, 2005–2007. Clusters in the proportion of aquatic habitats that contained larvae, utilizing the ten-cell unit (TCU) as the spatial unit of analysis, were assessed through the use of local Moran's I indicator of spatial association, with a first order queen neighborhood. Cluster significance was determined based on a normal distribution and corrected for multiple comparisons utilizing the false discovery rate procedure (as described in Data and Methods).

There was significant heterogeneity in the spatial distribution of the proportion of aquatic habitats with mosquito larvae, considering the TCU as the unit of analysis. Focusing on drains, significant clusters of high proportion of drains with *Anopheles* larvae were observed in TCUs located in the northern part of the study area (Mikocheni ward), in the western part (Kipawa ward), and in the eastern-most area (Kurasini ward; Figure 5). Mikocheni is very well serviced with a large network of drains, but they are mostly located along minor roads in residential areas and are covered with grass. The majority of settlements in the other two wards with high density of anopheline positive drains are unplanned, and drains are frequently blocked and not well maintained. Very few significant clusters of low proportion of *Anopheles* presence in drains were observed. However, clusters of low proportion of *Culex* presence in drains were widespread, and clusters of high proportion partially overlapped those found for *Anopheles* larvae presence.

Results from the drain assessment survey indicated almost complete absence of maintenance activities, common presence of waste materials inside the drains, inadequate water flow, occurrence of flooding during the rainy season, and presence of *Anopheles* and *Culex* larvae (Table 2). Important characteristics that impact the presence of *Anopheles* larvae in drains were related to water flow and vegetation occurrence (Table 3, Model 1). Compared with a drain segment where water was flowing at normal velocity, the odds of *Anopheles* larval presence in drain segments with water flowing at low velocity were 5.9 (95% CI 4.1–8.7) times larger, and those with stagnant water were 8.3 (95% CI 5.8–11.7) times larger. Drain segments with vegetation were 3.4 (95% CI 2.5–4.6) times more likely to contain *Anopheles* larvae. Finally, *Anopheles* larvae were more likely to occur in drain segments that also contained *Culex* larvae (OR = 18.3, 95% CI 13.4–25.0). An important difference between characteristics that impacted the presence of *Anopheles* and *Culex* larvae was related to the occurrence of accumulated waste materials in drains (Table 3, Model 2). There was a strong association between the presence of waste materials and the probability of finding *Culex* larvae (OR = 3.5, 95% CI 2.5–4.8).

**Table 2:** Summary statistics of surveyed drain segments.

Characteristics	Municipality		
	Kinondoni	Temeke	Ilala
Total number of drain segments	1,768	675	829
Total extension (km)	53.2	23.3	31.1
Total number of drains	172	81	85
Average extension per segment (m)	30.1	34.5	37.4
Average depth per segment (m)	0.5	0.7	0.7
Average width per segment (m)	1.0	1.2	1.2
Represented in the Cadastral maps (%)	39.2	26.1	8.2
Located in residential area (%)	89.9	92.4	80.3
Lined (%)	81.4	83.4	75.2
Open (%)	63.6	82.1	73.1
Constructed with cement slabs/blocks (%)	40.6	50.9	44.0
Constructed with concrete (%)	35.3	17.2	25.8
Accessible by truck (%)	84.1	67.9	78.8
Contained solid wastes (%)	46.6	66.8	48.5
Plastics (%)	29.3	51.2	29.9
Plastics and other garbage (%)	69.9	44.1	67.7
Water was stagnant (%)	27.7	8.4	26.9
Water was flowing at low velocity (%)	22.3	11.7	20.6
Water was flowing at normal velocity (%)	15.8	11.0	7.8
Dry segment (%)	33.1	67.8	44.6
Presence of human-made connection (%)	14.1	7.0	8.7
Contained *Culex* larvae (%)	19.4	12.3	12.9
Contained *Anopheles* larva (%)	18.6	6.5	12.6
Contained vegetation (%)	21.8	41.6	23.0
Cleaning efforts were being undertaken (%)	0.6	2.3	1.5
Surveyed in the dry season – Jan–Feb/Jun–Sep (%)	74.9	27.7	65.9
Presented history of flooding (%)	51.3	21.6	23.2

**Table 3:** Logistic regression models on the presence of larvae in drain segments.

	Model 1 - *Anopheles*				Model 2 - *Culex*			
	Odds ratio	95%	CI	p-value	Odds ratio	95%	CI	p-value
Drain segment contains a human-made connection	1.56	1.07	2.26	0.020	1.80	1.28	2.53	0.001
Drain segment is lined	0.86	0.60	1.24	0.429	1.00	0.71	1.41	0.990
Drain segment is located in a residential area	2.91	1.83	4.64	<0.001	0.37	0.25	0.55	<0.001
Drain segment contains waste materials	1.27	0.92	1.74	0.141	3.48	2.52	4.80	<0.001
Drain segment contains vegetation	3.39	2.50	4.61	<0.001	1.81	1.36	2.42	<0.001
Maintenance efforts to maintain the drain segment clear are regularly undertaken	0.08	0.02	0.38	0.002	4.69	2.13	10.36	<0.001
Drain segment has a history of flooding	0.76	0.55	1.05	0.092	1.99	1.46	2.72	<0.001
Water in the drain segment flows at low velocity	5.93	4.06	8.66	<0.001	2.32	1.62	3.32	<0.001
Water in the drain segment is stagnant	8.32	5.84	11.86	<0.001	5.55	3.95	7.80	<0.001
Drain segment accessible by a truck	1.23	0.84	1.79	0.293	0.50	0.35	0.70	<0.001
Drain segment surveyed during the dry season	1.20	0.85	1.69	0.305	0.78	0.56	1.08	0.131
Drain built before 1992	1.01	0.75	1.37	0.931	1.77	1.33	2.37	<0.001
Drain segment located in Temeke Municipality	0.42	0.25	0.73	0.002	1.66	1.05	2.63	0.029
Drain segment located in Kinondoni Municipality	1.35	0.93	1.96	0.110	1.63	1.12	2.38	0.011
Drain segment contains *Culex* larvae	18.31	13.43	24.95	<0.001				
Drain segment contains *Anopheles* larvae					17.95	13.25	24.32	<0.001
ROC	0.9249				0.9258			

Each covariate is binary, and therefore the absence of the condition they describe comprise the reference group. In the case of Municipality, Ilala is the reference group.

Model diagnostics indicated a good fit, as represented by a ROC value of approximately 0.93 (Table 3). In addition, a robust estimation did not change significantly the standard errors, and therefore heteroscedasticity was not likely to occur in the models. Sensitivity of the model was assessed by removing 11 observations with extreme values (large residuals) in Model 1 and 31 observations in Model 2. The new fitted models indicated no significant changes in the estimated coefficients.

## Discussion

Dar es Salaam is served by an extensive network of drains (approximately 1,130 km), designed to lower the water level in the city and to prevent the accumulation of stagnant water suitable for vector proliferation [25], [37]. In fact, some of these drains were specifically designed by anti-malarial engineers to drain water from malarious areas, and were locally known as ‘anti-malaria drains’ [40]. The current conditions of drains, however, increase the risk of vector breeding, and they commonly become associated with a different meaning: ‘malaria drains’. The results of our analysis provide evidence that drains are the most common aquatic habitat and the most common habitat containing *Anopheles* and *Culex* larvae in the city of Dar es Salaam. Nevertheless, drains seem not to be the most preferred habitat by these species, since a small proportion contained larvae. In contrast, the much fewer aquatic sites associated with urban agriculture are more likely to be found with larvae of both mosquito species. In addition, the vast majority of larval habitats in Dar es Salaam are human-made. Drains, borrow pits, and house foundations under construction comprised 55% of all habitats that contained *Anopheles* larvae and 69% of all open habitats that contained *Culex* larvae. These habitats are direct consequences of human actions, as opposed to puddles, swampy grounds, marshes and seepages which are mainly a result of the local environmental conditions.

In well maintained drain segments, where water can flow undisrupted and vegetation is absent, the likelihood of finding anopheline larvae was reduced by over 90%. Culicines showed a wider distribution in drain segments with various characteristics, particularly waste accumulation. Interestingly, there was a very strong positive association between the presence of anophelines and the presence of culicines. Similar findings have been reported from habitats in rural areas in East and West Africa [9], [65] indicating that there is no clear separation between ‘typical’ *Anopheles* and *Culex* larval habitats. These results highlight that, under ideal conditions, drains should serve as a tool for source reduction of all mosquito types, including the vectors of numerous neglected tropical diseases, and therefore it is not the habitat *per se* that is conducive to mosquito breeding, but human activity and lack of maintenance that can produce ideal conditions for larval development.

The typology of breeding habitats did vary spatially. Some habitat types, such as ponds, agricultural sites, and drains are located in specific areas, and are not randomly distributed across the city. In the case of agriculture, previous studies showed that the presence of larvae was more likely in larger fields (sizes between 100 and 400 m^2^), located in lowland areas, close to rivers or ponds but far from drains, and with loamy or clayey soils [22]. Some areas may lack drains because they are unplanned settlements or because their local ecology does not require modifications to reduce the water level. Significant clusters of TCUs with high proportion of drains with *Anopheles* and *Culex* larvae were observed in three out of the 15 surveyed city wards. This spatial variability is likely to be a result of idiosyncratic interactions between the local ecology and human behaviors that have the potential to minimize or augment the negative impacts of human-made transformations on vector development. Spatial exploratory approaches similar to those utilized in this analysis, as well as more sophisticated spatial modeling, should be applied more frequently in entomological studies to better capture the occurrence of local patterns in the distribution of positive habitats, and to shed light on the likely factors that determine such patterns.

A weakness of our survey is that TCUs were not distinguished by specific characteristics (e.g., land use and prevalence of unplanned settlements) or by their size (geographical area and residents) even though the spatial variation in habitat abundance is likely to be partially influenced by these variables. However, the clustering pattern demonstrated in our analysis is particularly important as a first step toward better understanding of the local variations of human exposure to LF and malaria, and could be utilized for more targeted interventions [66]–[68].

The majority of open vector breeding sites could be readily avoided by keeping the drain network in Dar es Salaam in good condition, through the implementation of routine EM activities, embedded in an integrated vector management (IVM) approach [69] targeting multiple vector-borne diseases. This would not only prevent drains to serve as breeding sites, but would also reduce the number of other aquatic habitats by draining high ground water level, which otherwise leads to pooling of stagnant water on swampy grounds in lowland areas frequently used for urban agriculture. Restoring the drains would also reduce UMCP costs by eliminating, on average, around 42% of all potential mosquito larval habitats that are currently treated with larvicides in weekly intervals (Table 1). In addition, it could potentially reduce other vector-borne and water-borne diseases, and contribute to the improvement of local environmental conditions. While initial EM activities focused on cleaning and repair may demand significant financial resources (given the current precarious conditions of drains in Dar es Salaam), they tend to have a short duration and are immediately followed by routine and much less expensive maintenance activities with long-lasting impacts [27], [70]–[73].

These activities would greatly benefit from involvement of community members [74]–[76], one of the characteristics of an IVM approach [69]. This would contribute to engage local residents, to develop a sense of ownership, to improve environmental responsibility among the population, to avoid further constraints to the currently insufficient health staff, and to ultimately facilitate actions toward poverty alleviation and sustainable development [74], [77]. In addition, efforts should include local capacity building [69], since much of the skilled personnel needed to properly plan, implement, monitor, and evaluate vector control interventions, including EM-related activities, is scarce in LF- and malaria-endemic countries. The establishment of multidisciplinary groups, bringing together entomologists, physicians, social scientists, biologists, engineers, hydrologists, and urban planners, could certainly improve vector control activities, and facilitate the dialogue and collaboration between different government sectors, that rarely work in partnership [78].

Such multidisciplinary and community-based approaches applied to contemporary EM interventions would represent an important distinction from historical EM efforts, which were mainly vertical (and often authoritarian) programs [31], [33], [79], [80]. Also, previous EM programs were heavily based on new engineering works, while contemporary programs demand a special attention to recover and maintain existing infra-structure (likely to be over utilized and deteriorated as a result of fast city growth and deficient maintenance). In addition, the planning, implementation and evaluation of routine sensitization and educational programs targeted to disseminate knowledge and to promote behavior change that could result in reduction of human-made vector breeding sites has seen little application in Africa [81], [82].

A community-based pilot EM intervention conducted in Dar es Salaam in 2008 provided initial evidence of the potential to increase awareness and local engagement after sensitization campaigns [73]. We argue that a sensitization effort should engage community leaders (taking advantage of the current neighborhood structure in Dar es Salaam, where each TCU has an elected leader), local community groups (e.g., religious groups), non-governmental organizations, school teachers, and local health officials. The use of local community members modestly paid to conduct vector control activities, including drain maintenance, could be expanded given that proper supervision is in place [56], [73]. Initiatives to be promoted by sensitization campaigns could include not only the dissemination of knowledge regarding mechanisms of disease transmission and prevention, but also the provision of information on ways to dispose waste, cultivate crops, build/renovate houses, and create sewage connections that minimize the risk of vector development. Further research and experiments are needed to evaluate the best sensitization program scope, structure, and targeted population to effectively promote behavior change to reduce the number of larval habitats.

Currently, there is a lack of synergy between the National Malaria Control Program (NMCP)/UMCP and the National Lymphatic Filariasis Elimination Program (NLFEP), although opportunities for combined action do exist. Strategies of the NMCP in Dar es Salaam include the introduction of malaria rapid diagnostic tests in health facilities, the distribution of long-lasting insecticidal nets, the improvement of intermittent preventive treatment of malaria in pregnancy, and wide access to artemisinin-based combination therapy [83]; the UMCP focuses on spraying larvicide in 15 wards, planned to be progressively scaled-up to all urban wards of Dar es Salaam. With regards to LF, the NLFEP began in 1997, focusing on mass drug administration (MDA), lymphoedema management, and hydrocelectomies (surgery for scrotal swellings) [5]. The first round of MDA was launched in the United Republic of Tanzania in 2000, using a combination of ivermectin (Mectizan®) and albendazole. The MDA conducted in Dar es Salaam in 2006–2007 covered approximately 65% of the population. Although vector control focusing on treated nets and reduction of mosquito breeding sites also comprise the list of the NLFEP recommended strategies, these activities are currently regarded as NMCP tasks, and LF control is heavily based on MDA. Nonetheless, MDA alone may not be sufficient to achieve LF elimination due to potential development of drug resistance, resource constraints, and operational difficulties to achieve high coverage in urban areas [3], [84]. These challenges become more critical considering that the drugs utilized on MDA efforts suppress the production of new microfilariae but do not kill adult worms, and therefore, the duration of the MDA should exceed the average longevity of adult worms, 5–10 years [85]. Implementation of vector control is critical to minimize transmission when MDA efforts have moderate coverage or are prematurely ceased.

Considering the importance of drains as larval habitats for both LF and malaria vectors, and the often constrained availability of financial resources in countries endemic to both diseases [4], [86], the need for integrated EM efforts stands out as crucial. Although our analysis focused on open habitats, a large number of culicines breed in closed habitats [53], and therefore the EM efforts here described are not the single solution to reduce LF vectors. Further studies are needed in order to investigate the dynamics of LF transmission by different mosquito species, originating from different habitats, as well as the potential importance of LF and malaria co-infections on levels of disease transmission [4]. Strategies to reduce larval development in closed habitats have been successfully adopted in Dar es Salaam and elsewhere [87], [88], and they need to be incorporated in an IVM approach [69], jointly planned and launched by LF and malaria control programs.

In 1997, the World Health Assembly called for LF elimination after the International Task Force for Disease Eradication identified the disease as potentially eradicable [89]. A decade later, a call for malaria eradication was made during the Gates Malaria Forum [90], [91]. A synergy between efforts to control each disease where they co-exist, identifying common strategies, combining monitoring activities, optimizing the use of limited financial resources, and carefully evaluating the cost-effectiveness of the joint venture can potentially contribute to successful outcomes, as well as provide important lessons for other potential concerted control efforts. Ultimately, an initiative to promote community-based EM and sensitization and educational programs, as part of a larger IVM approach that also targets open breeding habitats, is expected to optimize current efforts of the NMCP/UMCP and the NLFEP, mitigate some of the consequences of the current pace and pattern of urban growth [50], and add to the city's efforts to ensure environmental sustainability [92], as proposed by the Millennium Development Goal 7 (http://www.undp.org/mdg/goal7.shtml). Further studies are needed to provide definite evidence of such potential successful outcomes.
